# Prevention of postoperative proliferative vitreoretinopathy in complex retinal detachments with serial intravitreal methotrexate injections

**DOI:** 10.1186/s40942-026-00856-9

**Published:** 2026-04-22

**Authors:** Lucas Valadão de Brito Soares, Tiago Nelson de Oliveira Rassi, Wener Passarinho Cella, Mauricio Maia

**Affiliations:** 1 Hospital de Referência Oftalmológica - Vision One, Av. Jerônimo de Albuquerque, 540, HRO, Bequimão, São Luís, CEP: 65060-645 MA Brazil; 2 Federal University of São Paulo, UNIFESP/EPM-SP, São Paulo, Brazil; 3 Centro Universitário Unidade de Ensino Superior Dom Bosco, São Luís, Brazil

**Keywords:** Proliferative vitreoretinopathy, Rhegmatogenous retinal detachment, Intravitreal methotrexate, Pars plana vitrectomy, Retinal reattachment, Retinectomy

## Abstract

**Background:**

Proliferative vitreoretinopathy (PVR) remains the leading cause of surgical failure after rhegmatogenous retinal detachment (RRD) repair. Intravitreal methotrexate (MTX) has emerged as a potential antiproliferative adjunct; however, the available evidence remains inconclusive. This study evaluated the safety and clinical outcomes associated with a standardized protocol of serial intravitreal MTX injections administered throughout the critical postoperative period in eyes with complex RRD.

**Methods:**

This retrospective observational analysis included 49 eyes of 49 patients selected from 50 consecutively screened patients undergoing pars plana vitrectomy (PPV) for complex RRD, defined as PVR grade C, recurrent retinal detachment due to PVR, or retinal detachment associated with penetrating ocular trauma. All included eyes were treated according to a standardized institutional protocol consisting of an intraoperative intravitreal MTX injection (400 µg/0.1 mL), followed by biweekly injections through postoperative week 12. The primary outcome was single-surgery anatomic success (SSAS), defined as retinal reattachment without fibrotic membrane formation at 12 weeks. Secondary outcomes included final retinal reattachment over total follow-up, changes in best-corrected visual acuity (BCVA), and safety outcomes.

**Results:**

Forty-nine of 50 screened patients met the inclusion criteria and were included in the final analysis. SSAS at 12 weeks was achieved in 42 of 49 eyes (85.7%). Seven eyes required reoperation during follow-up, yielding a final retinal reattachment rate of 93.8% over the total follow-up period. Mean BCVA improved significantly from 2.52 ± 0.58 logMAR preoperatively to 1.23 ± 0.84 logMAR at 3 months (*p* < 0.001). Mean follow-up was 14.7 ± 10.8 months. The most frequent adverse event among analyzed eyes was conjunctival hyperemia, which resolved after completion of the MTX protocol. No severe ocular or systemic MTX-related adverse events were observed.

**Conclusions:**

Serial intravitreal MTX administered during the early postoperative period showed a favorable safety profile and was associated with encouraging anatomic and visual outcomes in this cohort of complex RRD. However, given the retrospective design, lack of a control group, cohort heterogeneity, potential selection bias, and the influence of adjunctive surgical procedures, these findings should be interpreted cautiously and as hypothesis-generating. Prospective controlled studies are required to better define the role of MTX in preventing postoperative PVR.

**Supplementary Information:**

The online version contains supplementary material available at 10.1186/s40942-026-00856-9.

## Introduction

Proliferative vitreoretinopathy (PVR) remains the leading cause of failure after rhegmatogenous retinal detachment (RRD) repair. Despite advances in vitreoretinal surgery, the incidence of clinically relevant PVR has remained relatively stable over time, and affected eyes continue to face a high risk of recurrent detachment and poor visual outcomes. Surgical management remains the cornerstone of treatment, often requiring pars plana vitrectomy (PPV), membrane peeling, internal tamponade, and, in selected cases, adjunctive procedures such as scleral buckling and retinectomy [1, 2].

Because surgery alone does not fully prevent postoperative fibrocellular proliferation, several pharmacologic adjuncts have been investigated, including corticosteroids, antimetabolites, anti-vascular endothelial growth factor agents, retinoic acid, and colchicine [3–6]. However, results have been inconsistent, and no pharmacologic strategy has become standard of care for routine prevention of postoperative PVR.

More recently, intravitreal methotrexate (MTX) has emerged as a potentially useful adjunct because of its antiproliferative and anti-inflammatory properties [7, 8] Intravitreal MTX has been used in ophthalmology for noninfectious uveitis, intraocular lymphoma, and, more recently, as an adjunct in eyes with established or high-risk PVR [8–11] Experimental and translational studies have also supported a biological rationale for MTX in inhibiting cellular proliferation relevant to PVR pathogenesis [12, 13].

Nevertheless, the clinical evidence remains inconclusive. Retrospective series have suggested favorable outcomes with serial postoperative MTX dosing, whereas studies using single intraoperative dosing have not consistently shown benefit [14, 15] More recent evidence has provided a more nuanced understanding of its role. In a multicenter randomized controlled trial, Nourinia et al. reported that repeated intra-silicone oil MTX injections did not significantly improve retinal reattachment rates, although they significantly reduced limited PVR recurrence and appeared safe [16]. In addition, a recent systematic review and meta-analysis by Kocak et al. found a clinically relevant but not statistically significant reduction in recurrent retinal detachment risk with adjunctive MTX, underscoring the need for larger, high-quality randomized trials [17].

The fibroproliferative response characteristic of PVR is believed to occur predominantly during the first 12 postoperative weeks, a period marked by cellular activation, migration, cytokine release, and extracellular matrix deposition [6, 18]. Because a single intravitreal MTX injection may not provide pharmacologic coverage throughout this interval, serial dosing has been proposed as a strategy to maintain therapeutic activity during the full period of biological risk [14, 19, 20].

Based on this rationale, the present study evaluated the safety and clinical outcomes associated with a standardized serial intravitreal MTX protocol administered throughout the early postoperative period in eyes undergoing PPV for complex RRD. The primary objective was to assess single-surgery anatomic success (SSAS) at 12 weeks. Secondary outcomes included final retinal reattachment over total follow-up, visual function, and safety.

## Methods

### Study design and ethics

This study was a retrospective observational analysis of consecutive patients treated in routine clinical practice according to a standardized institutional MTX protocol. Although written informed consent for treatment and data use was obtained before enrollment, the present investigation was not conducted as a prospective interventional trial. Rather, outcome data were analyzed retrospectively from electronic medical records after completion of clinical care. The study protocol was approved by the institutional Data Review Committee, and all procedures adhered to the tenets of the Declaration of Helsinki.

### Participants

We screened 50 consecutive patients who underwent PPV for RRD between February 2020 and April 2025. Cases were classified as complex retinal detachment if at least one of the following criteria was present: PVR grade C, recurrent retinal detachment due to PVR, or retinal detachment associated with penetrating ocular trauma. These diagnostic categories were grouped because they all represent complex detachments at elevated risk of postoperative proliferative complications, which was the population of interest for evaluating the safety and feasibility of the serial MTX protocol.

To be included in the final analysis, eyes were required to have completed at least seven intravitreal MTX injections according to the study protocol and to have a minimum postoperative follow-up of 12 weeks. Eyes with incomplete follow-up or fewer than seven MTX administrations were excluded from the final analysis.

All patients were examined and operated on by the same vitreoretinal surgeon (L. V. B. S.) at a single tertiary referral eye care center (Hospital de Referência Oftalmológica, HRO). PVR severity was classified according to the updated Retina Society Classification (1991) [21].

Collected data included demographic information, preoperative ocular history, surgical details, and postoperative findings. Best-corrected visual acuity (BCVA) was measured preoperatively and postoperatively using a Snellen chart and subsequently converted to logarithm of the minimum angle of resolution (logMAR) units for statistical analysis. Postoperative evaluation included optical coherence tomography (OCT) when clinically feasible.

### Surgical procedure

All surgeries were performed using a 25-gauge Constellation Vision System (Alcon Laboratories Inc., Fort Worth, TX, USA). In phakic eyes, combined phacoemulsification with intraocular lens implantation was performed during the same surgical session.

A standard bimanual pars plana vitrectomy with scleral depression was carried out to achieve meticulous vitreous base shaving, including removal of the anterior vitreous. Endolaser photocoagulation was applied to all identified retinal breaks. Scleral buckle placement (model S2971, style #42; Labtician Ophthalmics) was performed at the surgeon’s discretion in cases exhibiting significant anterior retinal shortening.

Intraoperative adjuncts were used to facilitate tissue visualization. Triamcinolone acetonide was used in all cases to aid identification of the posterior hyaloid and was not part of the postoperative antiproliferative protocol under investigation. Brilliant blue G was used for visualization of the internal limiting membrane (ILM), and trypan blue was used for staining of preretinal membranes associated with PVR, when indicated. When ILM striae were observed, macular peeling was performed, extending to the vascular arcades. In cases of preretinal membranes located in the mid-periphery or peripheral retina, membrane peeling was carried out using 27-gauge forceps.

Perfluorocarbon liquid (PFCL) was used intraoperatively to assist retinal flattening. If retinal contraction persisted despite membrane removal and PFCL infusion, intraoperative retinectomy was performed using endodiathermy and scissors. Following retinal reattachment under PFCL, a fluid-gas exchange was performed, and a final internal tamponade was achieved with silicone oil (Oxane 5700; Bausch & Lomb).

### Methotrexate protocol

At the conclusion of surgery, methotrexate (Tevametho 50 mg/2 mL; Teva Farmacêutica) was injected into the silicone oil tamponade at a dose of 400 µg/0.1 mL. Additional intravitreal MTX injections at the same dose were administered every two weeks through postoperative week 12, for a total of seven doses, provided no contraindications emerged during follow-up.

This serial postoperative dosing schedule was informed by previously published MTX protocols designed to maintain antiproliferative activity during the early postoperative period, when PVR development is most likely to occur [14, 19].

### Outcomes

The primary outcome was SSAS, defined as retinal reattachment without evidence of fibrotic membrane formation at 12 postoperative weeks. This composite definition was adopted to capture not only anatomical reattachment but also the absence of clinically significant proliferative activity within the treatment window. The authors acknowledge that this definition differs from the more conventional use of the term in the literature, which typically refers to retinal reattachment after a single surgical procedure without specifying a fixed time point or incorporating membrane formation as a component (see Discussion). Secondary outcomes included final retinal reattachment over the total follow-up period, changes in BCVA, intraocular pressure, and postoperative adverse events.

### Statistical analysis

Data were initially analyzed using descriptive statistics. Categorical variables were summarized as absolute and relative frequencies, while continuous variables were expressed as means, standard deviations, medians, and interquartile ranges, as appropriate.

Associations between categorical variables were evaluated using Fisher’s exact test. Linear mixed-effects regression models were employed to assess the effects of diagnosis and intraoperative retinectomy on changes in visual acuity over time. When statistically significant differences were identified, post hoc comparisons were performed using Wald tests with Bonferroni correction.

The assumption of normality for continuous variables was assessed using the Kolmogorov-Smirnov test. A two-sided significance level of 5% was adopted for all analyses. Statistical analyses were performed using SPSS software version 20.0 (IBM Corp., Armonk, NY, USA) and Stata software version 18 (StataCorp LLC, College Station, TX, USA).

## Results

Of 50 consecutively screened patients, 49 eyes of 49 patients met the inclusion criteria and were included in the final analysis. One screened patient was excluded because the minimum number of seven intravitreal MTX injections was not completed owing to significant discomfort during the injection procedure. The clinical course of this excluded patient is described in the Safety Outcomes section and illustrated in Fig. 2a and d.

The mean patient age was 58.5 ± 13.6 years. Demographic and baseline ocular characteristics are summarized in Table 1. Overall, 32 patients (65.3%) were male, and 34 eyes (69.4%) presented with PVR grade C. Recurrent retinal detachment due to PVR was observed in 9 eyes (18.4%), and 6 eyes (12.2%) were associated with penetrating ocular trauma. Intraoperative retinectomy was performed in 8 eyes (16.3%). The mean follow-up duration was 14.7 ± 10.8 months, with a median of 12.4 months (IQR, 5.1–22.3).

**Table 1:** Demographic and ocular characteristics of the study population

Variable	*n* (%) or Mean ± SD	Median (IQR)
Sex		
Female	17 (34.7)	–
Male	32 (65.3)	–
Age (years)	58.5 ± 13.6	60.0 (50.5–66.5)
Diagnosis		
PVR grade C	34 (69.4)	–
Re-detachment due to PVR	9 (18.4)	–
Penetrating ocular trauma	6 (12.2)	–
Intraoperative retinectomy		
No	41 (83.7)	–
Yes	8 (16.3)	–
Follow-up time (months)	14.7 ± 10.8	12.4 (5.1–22.3)

**Abbreviations**: PVR, proliferative vitreoretinopathy; IQR, interquartile range (P25–P75)

Pars plana vitrectomy was combined with scleral buckle placement in 38 of 49 eyes (77.5%), with an SSAS rate of 86.8% in this subgroup. In the remaining 11 eyes undergoing vitrectomy without adjunctive scleral buckle, the SSAS rate was 81.8%. Because buckle status was not part of the original analytical framework, outcomes were not formally stratified according to postoperative anatomical status. All included eyes received silicone oil tamponade (5000 cSt), with no evidence of silicone oil-related inflammation or toxicity. The first intravitreal MTX injection (400 µg/0.1 mL) was administered intraoperatively immediately after silicone oil placement, followed by biweekly injections through the 12th postoperative week, for a total of seven doses.

### Anatomic outcomes

**Fig. 1:**
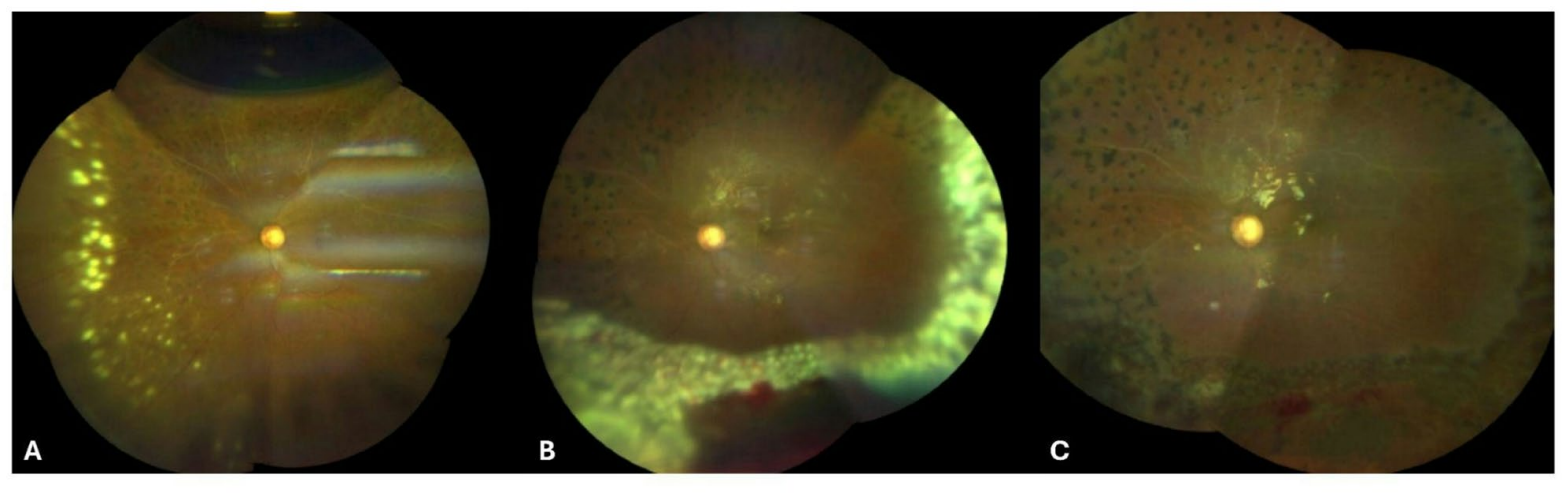
Representative case of primary rhegmatogenous retinal detachment with an inferior horseshoe tear, initially treated with pars plana vitrectomy and 20% sulfur hexafluoride (SF₆) gas tamponade, in a patient with a history of superior hemispheric retinal vein occlusion and prior superior laser photocoagulation. **a.** Fundus photograph obtained 10 days after the first surgery, showing initial postoperative appearance. **b.** Recurrent retinal detachment due to PVR, managed with surgical reintervention two months after the initial vitrectomy; image shows the postoperative appearance two weeks after retinectomy (see supplementary surgical video: https://vimeo.com/1110501077/41bc90a169?share=copy. **c.** Fundus photograph obtained 12 weeks after reintervention, demonstrating adequate retinectomy healing with stable retinal attachment and no evidence of fibrotic membrane formation

The primary endpoint, SSAS, defined as retinal reattachment at 12 weeks without evidence of fibrotic membrane formation (Fig. 1a and c), was achieved in 42 of 49 eyes (85.7%). Seven eyes underwent reoperation during follow-up because of postoperative contractile membrane formation and/or recurrent tractional redetachment. All seven reoperations were performed during the 12-week MTX treatment period, and all patients completed the protocol. The median interval from primary surgery to reoperation was 6 weeks (range, 3–9 weeks). Final retinal reattachment over the total follow-up period was achieved in 46 of 49 eyes (93.8%). Three eyes exhibited persistent localized PVR without macular involvement despite reoperation.

Subgroup analysis showed SSAS rates of 88.2% (30/34) in eyes with PVR grade C, 88.9% (8/9) in eyes with recurrent retinal detachment due to PVR, and 66.7% (4/6) in eyes with traumatic retinal detachment.

The distribution of SSAS according to diagnosis and intraoperative retinectomy status is shown in Table 2. No statistically significant association was found between SSAS and diagnostic category (*p* = 0.355) or between SSAS and performance of intraoperative retinectomy (*p* = 0.581).

**Table 2:** Single-surgery anatomic success according to diagnosis and retinectomy

Variable	SSAS – No *n* (%)	SSAS – Yes *n* (%)	*p* value
Overall	7/49 (14.3)	42/49 (85.7)	–
Diagnosis			0.355
PVR grade C	4/34 (11.8)	30/34 (88.2)	
Re-detachment due to PVR	1/9 (11.1)	8/9 (88.9)	
Penetrating ocular trauma	2/6 (33.3)	4/6 (66.7)	
Intraoperative retinectomy			0.581
No	7/41 (17.1)	34/41 (82.9)	
Yes	0/8 (0.0)	8/8 (100.0)	

*p* values obtained using Fisher’s exact test

**Abbreviation**: SSAS, single-surgery anatomic success

### Visual outcomes

**Chart 1:**
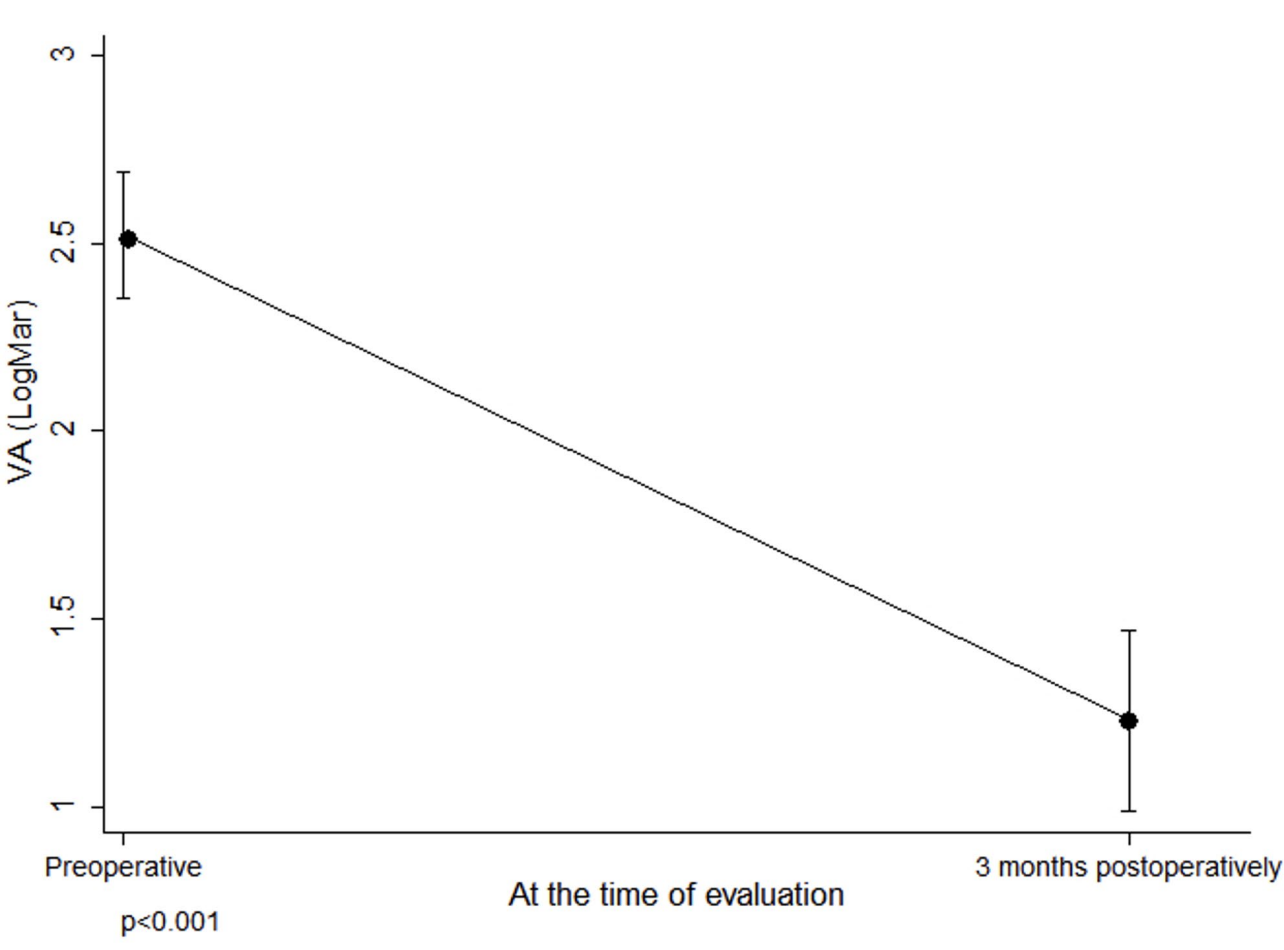
Mean best-corrected visual acuity (BCVA), expressed in logarithm of the minimum angle of resolution (logMAR), at baseline and 3 months postoperatively. Error bars represent 95% confidence intervals

**Chart 2:**
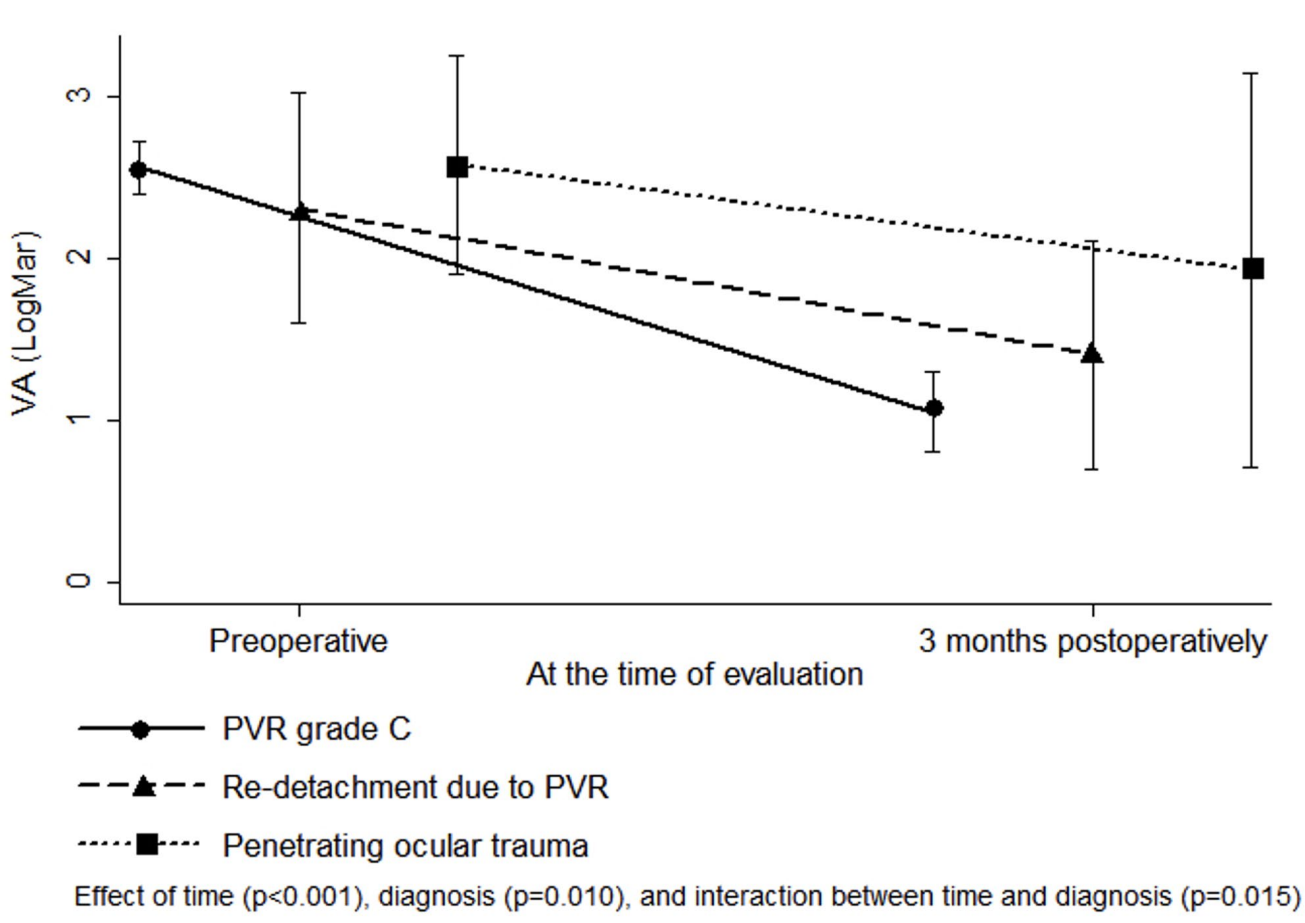
Mean best-corrected visual acuity (BCVA), expressed in logMAR, at baseline and 3 months postoperatively, stratified by diagnosis (PVR grade C, recurrent retinal detachment due to PVR, and penetrating ocular trauma). Error bars represent 95% confidence intervals

Overall, mean BCVA improved significantly at 3 months postoperatively (*p* < 0.001), from 2.52 ± 0.58 logMAR (approximate Snellen equivalent 20/8000) preoperatively to 1.23 ± 0.84 logMAR (approximate Snellen equivalent 20/350). Mean BCVA values and corresponding 95% confidence intervals over time are illustrated in Chart 1, with subgroup analysis by diagnosis shown in Chart 2.

**Chart 3:**
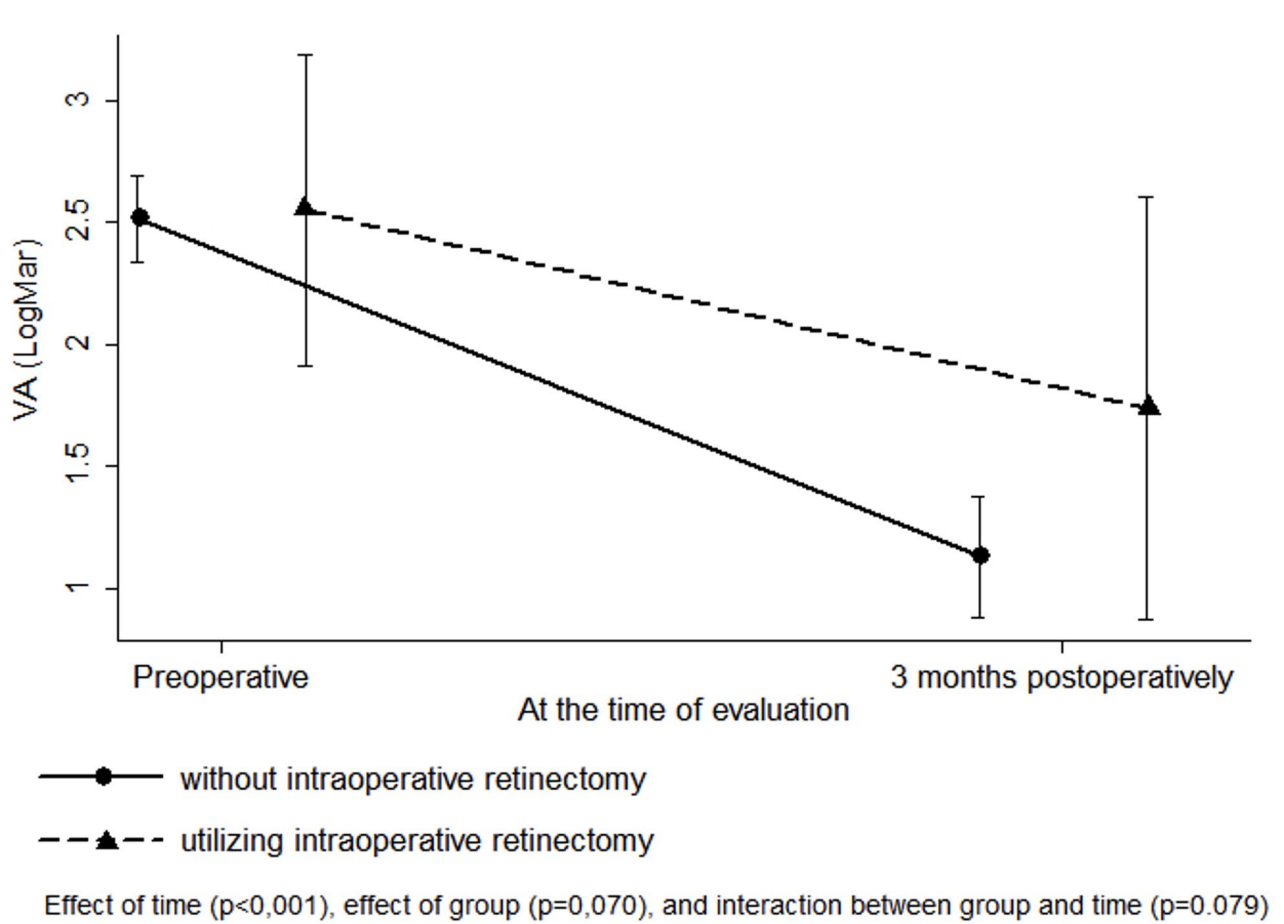
Mean best-corrected visual acuity (BCVA), expressed in logMAR, at baseline and 3 months postoperatively, according to intraoperative retinectomy status. Error bars represent 95% confidence intervals

Eyes undergoing intraoperative retinectomy achieved 100% SSAS. However, these cases demonstrated a trend toward poorer functional recovery, with a final mean BCVA of 1.74 logMAR (approximate Snellen equivalent 20/1200), as depicted in Chart 3. Visual acuity improved in 45 of 49 eyes and remained stable in the remaining cases. Detailed visual acuity changes stratified by diagnosis and retinectomy status are provided in Appendix 1.

### Intraocular pressure and postoperative complications

Mean intraocular pressure (IOP) during follow-up was 15.5 mmHg (median, 16.7 mmHg). One patient developed sustained IOP elevation beginning in the second postoperative month, which was refractory to topical hypotensive therapy. This patient subsequently underwent silicone oil removal combined with glaucoma valve implantation three months after the initial surgery.

Postoperative hypotony, defined as an IOP below 6 mmHg, was observed in three eyes (6.1%). All hypotonic eyes maintained anatomic retinal attachment and did not develop subsequent PVR.

Epiretinal membrane formation was observed in six eyes (12.2%), with only one case associated with foveal distortion requiring secondary membrane peeling. Cystoid macular edema occurred in three eyes (6.1%) and resolved with topical anti-inflammatory therapy, without the need for intravitreal corticosteroid injections. The distribution of postoperative complications is summarized in Table 3.

**Table 3:** Postoperative complications and ocular surface findings

Variable	*n* (%)
Epiretinal membrane	
No	43 (87.8)
Yes	6 (12.2)
Cystoid macular edema	
No	46 (93.9)
Yes	3 (6.1)
Hypotony	
No	46 (93.9)
Yes	3 (6.1)
Corneal changes	
Epithelial defect	1 (2.0)
Conjunctival hyperemia	49 (100.0)

### Safety outcomes

Among analyzed eyes, conjunctival hyperemia was frequently observed during the postoperative period and resolved after completion of the MTX injection protocol. A single case of corneal epithelial defect occurred during the third postoperative week and resolved with therapeutic contact lens placement, without interruption of subsequent MTX administrations. No severe ocular or systemic adverse events related to methotrexate were observed during the follow-up period.

**Fig. 2:**
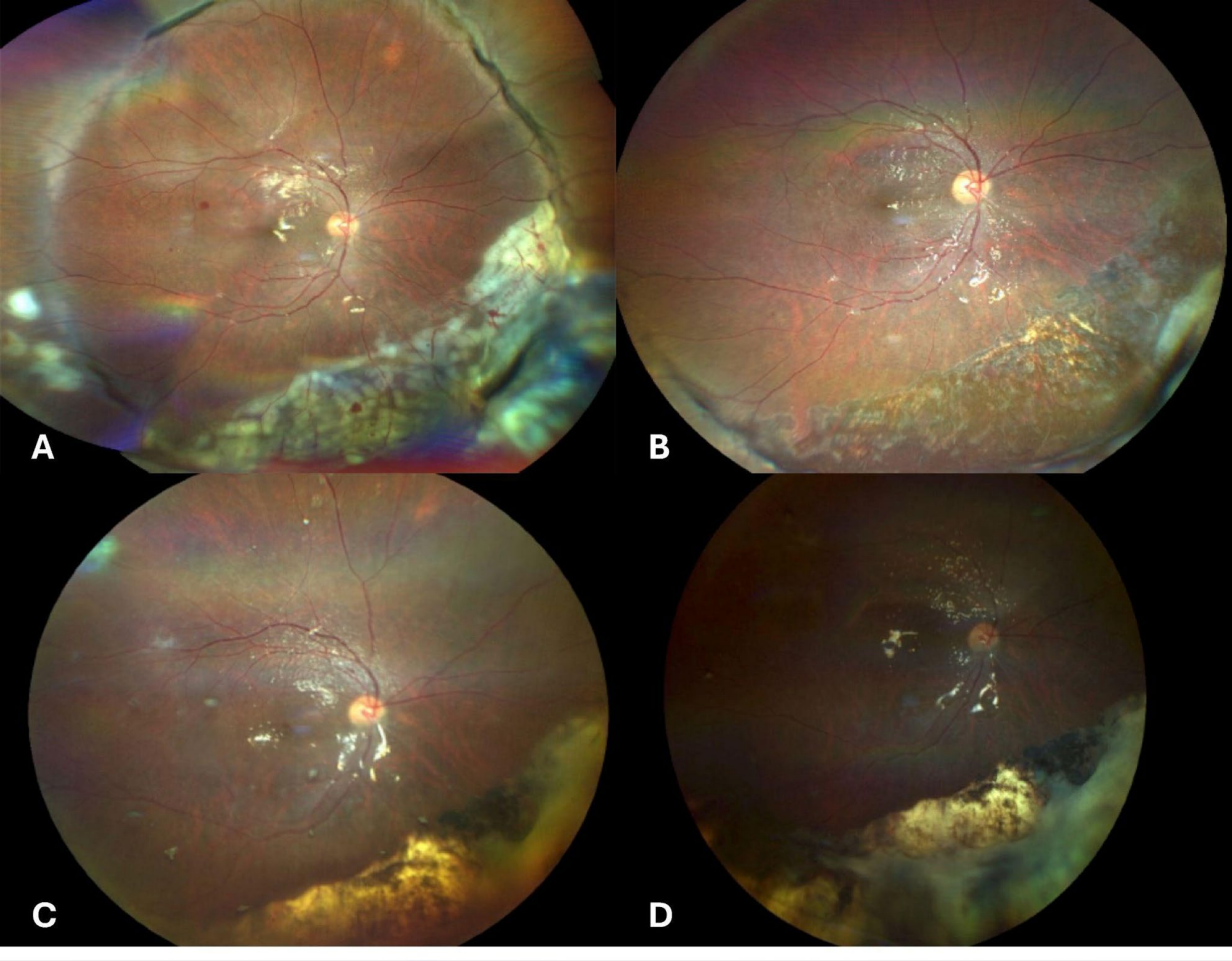
Representative case of late fibrotic membrane formation associated with anterior proliferative vitreoretinopathy and subsequent hypotony, occurring after discontinuation of methotrexate treatment at postoperative week 4. **a.** Second postoperative week, showing an attached retina without evidence of preretinal membranes. **b.** Fourth postoperative week, demonstrating well-pigmented laser scars and absence of preretinal membrane formation. **c.** Eighth postoperative week, with an attached retina and early fibrotic membrane development. **d.** Twelfth postoperative week, showing progression of fibrotic membranes associated with ocular hypotony

One additional screened patient, who was excluded from the final analysis because fewer than seven injections were completed, experienced significant discomfort during the week-4 injection and elected to discontinue treatment (Fig. 2a and d). This patient subsequently developed progressive anterior PVR with fibrotic membrane formation by week 8, followed by clinically significant hypotony by week 12, illustrating the natural history of the disease when the MTX protocol was not completed.

## Discussion

This study describes the outcomes of a consecutive cohort of eyes with complex rhegmatogenous retinal detachment treated with a standardized protocol of serial intravitreal methotrexate during the early postoperative period. In this retrospective single-arm series, the protocol showed a favorable safety profile and was associated with encouraging anatomic and visual outcomes. However, the present results should be interpreted as descriptive findings in a treated cohort rather than as evidence of therapeutic efficacy.

The absence of a control group is a major limitation. Without a contemporaneous or historical comparison cohort managed with surgery alone, it is not possible to determine the extent to which the observed outcomes were related to MTX itself, as opposed to other factors such as case selection, surgical technique, surgeon experience, or the use of adjunctive procedures. Accordingly, cross-study comparisons should be interpreted cautiously and are provided for context only.

The current findings should also be viewed in light of the evolving literature on MTX for PVR. Serial postoperative MTX regimens have previously been associated with favorable outcomes in retrospective reports, particularly when repeated dosing was used during the early proliferative period [14, 19, 20]. In contrast, single-dose intraoperative protocols have not consistently shown benefit [15]. More recent randomized evidence has further refined this interpretation. Nourinia et al. reported that repeated intra-silicone oil MTX injections did not significantly improve retinal reattachment rates, although they significantly reduced limited PVR recurrence and appeared safe [16]. Similarly, Kocak et al., in a systematic review and meta-analysis, found a clinically relevant but not statistically significant reduction in recurrent retinal detachment risk with adjunctive MTX [17]. Taken together, these data suggest that MTX remains a promising but unproven adjunctive strategy.

In the present cohort, SSAS at 12 weeks was 85.7%, and final retinal reattachment over total follow-up was 93.8%. These findings are encouraging, but they should not be interpreted as isolating the effect of MTX. The cohort was heterogeneous and included eyes with PVR grade C, recurrent detachment due to PVR, and retinal detachment associated with penetrating ocular trauma, all of which differ in biological behavior and prognosis [22]. Although these entities were grouped because they all represent complex detachments at high risk of postoperative proliferative complications, subgroup-specific findings should be considered exploratory, as the study was not powered for formal comparative subgroup analysis.

Interpretation is further complicated by the extent of surgical intervention. Many eyes underwent combined scleral buckling, membrane peeling, ILM peeling, or retinectomy according to intraoperative findings. These procedures may have independently influenced the observed anatomical outcomes. Notably, all eyes undergoing intraoperative retinectomy achieved SSAS, although functional recovery in this subgroup was more limited. This pattern is consistent with the established role of retinectomy in relieving severe traction while also reflecting the greater baseline complexity of such cases.

The definition of SSAS used in this study—retinal reattachment without fibrotic membrane formation at 12 postoperative weeks—merits specific discussion. This composite definition differs from the conventional use of the term in the retinal detachment literature, where SSAS typically refers to retinal reattachment after a single surgical procedure without specifying a fixed time point or incorporating membrane formation as a component. The more stringent definition adopted herein was chosen to capture not only anatomical reattachment but also the absence of clinically significant proliferative activity within the MTX treatment window. However, this non-standard definition limits direct comparison with previously published single-surgery success rates and should be considered when interpreting our results.

From a functional standpoint, BCVA improved significantly at 3 months. This finding is clinically relevant but should likewise be interpreted cautiously, given the absence of a comparator group and the heterogeneity of baseline disease severity. Although postoperative anatomical stability may contribute to visual improvement, the present design does not allow attribution of functional benefit specifically to MTX.

The safety profile observed in this cohort was favorable. No severe ocular or systemic MTX-related adverse events were observed among analyzed eyes. Conjunctival hyperemia was common during the treatment period and resolved after completion of the injection protocol. A single corneal epithelial defect resolved with conservative management. These findings are consistent with prior reports suggesting acceptable ocular tolerability of intravitreal MTX at currently used doses [10, 11, 16].

The relatively low rate of postoperative hypotony in this cohort is reassuring; however, any mechanistic interpretation should remain cautious. Although suppression of postoperative proliferative activity could hypothetically contribute to preservation of ciliary body function, the present study was not designed to evaluate this mechanism, and no causal inference can be made from these data.

It should also be acknowledged that the routine intraoperative use of triamcinolone acetonide for posterior hyaloid visualization may represent an additional confounding factor. Although triamcinolone was used strictly as a surgical aid and was largely removed during fluid-air exchange, residual anti-inflammatory effects during the early postoperative period cannot be entirely excluded.

Several limitations must be acknowledged. First, the retrospective, non-randomized design precludes definitive causal inference. Second, the absence of a control group limits interpretation of treatment effect. Third, the requirement for completion of at least seven MTX injections and adequate follow-up may have introduced selection bias by preferentially retaining patients with greater treatment tolerance and protocol adherence. Fourth, the study was conducted at a single center, with all surgeries and postoperative assessments performed by a single surgeon, and postoperative evaluations were unmasked, which may introduce bias. Fifth, the cohort was clinically heterogeneous and underwent varied adjunctive surgical maneuvers, making it difficult to isolate the contribution of any single intervention. Sixth, scleral buckle status was not formally analyzed by postoperative outcome subgroup. Finally, although the primary endpoint was assessed at 12 weeks to correspond to the planned duration of the serial MTX protocol and the early proliferative window of PVR, this time point may not fully capture late recurrence or delayed postoperative complications, even though the mean total follow-up exceeded 1 year.

Prospective controlled studies using harmonized endpoint definitions, adequate statistical power, and longer follow-up are needed to better define the role of serial intravitreal MTX in the prevention of postoperative PVR after complex retinal detachment repair.

## Conclusion

Serial intravitreal methotrexate administered during the early postoperative period was safe and was associated with favorable anatomic and functional outcomes in this cohort of complex rhegmatogenous retinal detachment cases. However, given the retrospective design, lack of a control group, cohort heterogeneity, potential selection bias, and the influence of adjunctive surgical procedures, these findings should be interpreted cautiously and as hypothesis-generating rather than confirmatory evidence of treatment efficacy. Prospective controlled studies are needed to better define the role of MTX in preventing postoperative proliferative vitreoretinopathy.

## Appendix

**Appendix 1:** Summary of best-corrected visual acuity (BCVA), expressed in logarithm of the minimum angle of resolution (logMAR), measured preoperatively and at 3 months postoperatively for the entire cohort and stratified by diagnosis and intraoperative retinectomy status. Values are presented as mean ± standard deviation. *PO* = postoperative. *p* values were derived from linear mixed-effects regression models

Group	Preoperative	3 months PO	Change (Post – Pre)	*p* (time)	*p* (group)	*p* (interaction)
Total (*n* = 49)	2.52 ± 0.58	1.23 ± 0.84	–1.29 ± 0.88	< 0.001	–	–
Diagnosis				< 0.001	0.019	0.015
PVR C (*n* = 34)	2.57 ± 0.46	1.06 ± 0.70	–1.51 ± 0.80			
Re-detachment (*n* = 9)	2.31 ± 0.93	1.41 ± 0.92	–0.90 ± 0.88			
Trauma (*n* = 6)	2.59 ± 0.64	1.93 ± 1.16	–0.65 ± 0.95			
Retinectomy				< 0.001	0.070	0.079
No (*n* = 41)	2.52 ± 0.56	1.13 ± 0.77	–1.39 ± 0.83			
Yes (*n* = 8)	2.55 ± 0.76	1.74 ± 1.04	–0.81 ± 1.02			

## Electronic Supplementary Material

Below is the link to the electronic supplementary material.

Supplementary Material 1

Supplementary Material 2

## Data Availability

The datasets used and/or analyzed during the current study are available from the corresponding author upon reasonable request.

## Abbreviations

BCVA: Best-corrected visual acuity
CI: Confidence interval
ILM: Internal limiting membrane
IOP: Intraocular pressure
IQR: Interquartile range
logMAR: Logarithm of the minimum angle of resolution
MTX: Methotrexate
OCT: Optical coherence tomography
PFCL: Perfluorocarbon liquid
PO: Postoperative
PPV: Pars plana vitrectomy
PVR: Proliferative vitreoretinopathy
RRD: Rhegmatogenous retinal detachment
SSAS: Single-surgery anatomic success
