# Supplementary material for: Prevention of postoperative proliferative vitreoretinopathy in complex retinal detachments with serial intravitreal methotrexate injections
Source: Int J Retina Vitreous. 2026 Apr 22;12:83. doi: 10.1186/s40942-026-00856-9 (PMC13251097; doi:10.1186/s40942-026-00856-9)
Supplement: Supplementary file 2 — Supplementary Material 2 [file 40942_2026_856_MOESM2_ESM.docx]

## Appendix 2. INFORMED CONSENT TO PARTICIPATE IN RESEARCH

| **Study Title:** Prevention of Postoperative Proliferative Vitreoretinopathy in Complex Retinal Detachments with Serial Intravitreal Methotrexate Injections  **Principal Investigator:** Lucas Valadão de Brito Soares, MD  **Institution:** Hospital de Referência Oftalmológica – Vision One, São Luís, Brazil  **Contact:** lucasvaladao3@gmail.com \| Av. Jerônimo de Albuquerque, 540, HRO, Bequimão, CEP: 65060-645, São Luís, MA, Brazil  **Ethics Committee Approval:** Hospital São Domingos Ethics Committee |
| --- |

## 1. PURPOSE OF THE STUDY

## You are being invited to participate in a scientific research study. This study aims to evaluate the safety and effectiveness of serial intravitreal injections of methotrexate (MTX) — a medication administered directly into the eye — for the prevention of postoperative proliferative vitreoretinopathy (PVR) in patients undergoing vitreoretinal surgery for complex retinal detachment.

## PVR is a serious complication that can occur after retinal detachment surgery and is the leading cause of surgical failure. This study seeks to determine whether serial MTX injections during the critical postoperative period can reduce the risk of this complication and improve surgical outcomes.

## 2. ELIGIBILITY

## You are eligible to participate in this study if you are undergoing pars plana vitrectomy (PPV) for complex retinal detachment, defined by the presence of PVR grade C, recurrent retinal detachment, or retinal detachment associated with penetrating ocular trauma.

## 3. STUDY PROCEDURES

## If you agree to participate, the following procedures will be performed:

## • Surgical procedure: You will undergo pars plana vitrectomy (PPV) as clinically indicated for your retinal detachment.

## • Intraoperative injection: At the conclusion of surgery, a single intravitreal injection of methotrexate (400 µg/0.1 mL) will be administered into your eye.

## • Postoperative injections: Additional intravitreal MTX injections will be administered every two weeks for up to 12 weeks after surgery (approximately 6 additional injections).

## • Clinical assessments: You will undergo regular follow-up visits including measurement of best-corrected visual acuity (BCVA), intraocular pressure (IOP), and ophthalmic examination.

## 4. POTENTIAL RISKS AND DISCOMFORTS

## Intravitreal methotrexate injections are generally well tolerated. The most commonly reported adverse event in this protocol is conjunctival hyperemia (redness of the eye), which is typically mild and resolves after completion of the injection series. Other potential risks associated with intravitreal injections include, but are not limited to: eye pain or discomfort, increased intraocular pressure, infection (endophthalmitis), vitreous hemorrhage, retinal detachment, and cataract formation. No severe ocular or systemic MTX-related adverse events have been reported at the doses used in this study. You will be monitored closely at each visit, and any adverse events will be managed promptly.

## 5. POTENTIAL BENEFITS

## You may benefit from participation in this study through a reduction in the risk of postoperative PVR and improved retinal reattachment rates. However, participation in this study does not guarantee a successful outcome. The results of this study may also contribute to improved care for future patients with complex retinal detachment.

## 6. CONFIDENTIALITY

## All information collected during this study will be kept strictly confidential. Your personal data will be stored securely and will only be accessible to the research team and the Ethics Committee. Your identity will not be disclosed in any publication or presentation. Data will be reported in aggregated form only, and no individually identifiable information will be published.

## 7. VOLUNTARY PARTICIPATION AND RIGHT TO WITHDRAW

## Your participation in this study is entirely voluntary. You are free to refuse to participate or to withdraw your consent at any time without penalty or loss of benefits to which you are otherwise entitled. Your decision will not affect the standard of medical care you receive. If you withdraw, any data collected before your withdrawal may still be used in the study analysis unless you specifically request its removal.

## 8. ETHICAL STANDARDS

## This study has been conducted in accordance with the ethical principles outlined in the Declaration of Helsinki (World Medical Association, 2013) and has been reviewed and approved by the Ethics Committee of Hospital São Domingos. All procedures comply with applicable local and national research regulations.

## 9. QUESTIONS AND ADDITIONAL INFORMATION

## Before signing this form, please feel free to ask any questions about the study. If you have questions at any time during the study, you may contact the principal investigator, Lucas Valadão de Brito Soares, MD, by email at lucasvaladao3@gmail.com or at the address listed above.

## PARTICIPANT CONSENT DECLARATION

## I, the undersigned, declare that:

## (a) I have read and understood the information contained in this Informed Consent Form;

## (b) I have had the opportunity to ask questions and have received satisfactory answers;

## (c) I understand that my participation is voluntary and that I may withdraw at any time without penalty;

## (d) I understand that my personal information will be kept confidential;

## (e) I freely and voluntarily consent to participate in the research study described above.

## ___________________________________________________ Signature of Participant

## Full Name (print): ____________________________________________

## Date: _____ / _____ / _________

## ___________________________________________________ Signature of Principal Investigator

## Name: Lucas Valadão de Brito Soares, MD

## Date: _____ / _____ / _________

## Witness (if applicable):

## ___________________________________________________ Signature of Witness

## Full Name (print): ____________________________________________

## Date: _____ / _____ / _________

## *This consent form has been prepared in accordance with the ethical guidelines of the International Journal of Retina and Vitreous (BioMed Central / Springer Nature) and the Declaration of Helsinki. One copy of this signed form will be provided to the participant, and one copy will be retained by the research team.*
